# Long-Lived Plasma Cells in Autoimmunity: Lessons from B-Cell Depleting Therapy

**DOI:** 10.3389/fimmu.2013.00494

**Published:** 2013-12-27

**Authors:** Matthieu Mahévas, Marc Michel, Jean-Claude Weill, Claude-Agnès Reynaud

**Affiliations:** ^1^INSERM UMR783, Faculté de Médecine-Site Broussais, Université Paris Descartes, Paris, France; ^2^INSERM U955, Établissement français du sang (EFS) Île-de-France, Hôpital Henri-Mondor, Créteil, France; ^3^Service de Médecine Interne, Centre de référence des cytopénies auto-immunes de l’adulte, Hôpital Henri-Mondor, Assistance Publique Hôpitaux de Paris, Université Paris-Est Créteil, Créteil, France

**Keywords:** rituximab, plasma cell niche, BAFF/Blys, belimumab, autoreactive antibody

## Abstract

A large number of auto-immune diseases are treated with rituximab, an antibody against CD20 that depletes most of the B-cells in the organism. The response to this treatment depends largely on the disease and the type of lymphoid cells involved in the auto-immune process. We recently reported that B-cell depletion in immune thrombocytopenia induced the appearance of pathogenic long-lived plasma cells in the spleen, which were not present before treatment or in non-auto-immune conditions. The spleen of treated patients produced an excess of the cytokine B-cell activating factor, which in *in vitro*-cultured splenic cells, could increase the longevity of plasma cells. Our results suggested that, paradoxically, the B-cell depletion itself, by altering the splenic milieu, promoted the differentiation of short-lived auto-immune plasma cells into long-lived ones. We describe the cellular and cytokinic components of the splenic plasma cell niche, notably CD4^+^ T cells and discuss possible survival factors that could be targeted simultaneously with rituximab-mediated B-cell depletion to interfere with plasma cell persistence.

## B-Cell Depletion, from Mice to Humans

For the last decade, anti-CD20-induced B-cell depletion has been increasingly used to treat several auto-immune conditions such as rheumatoid arthritis, vasculitis, and immune thrombocytopenia (ITP), but the clinical results have been disappointing (1–4). Moreover, mouse models of B-cell depletion have added to the confusion by showing incomplete B-cell depletion in lymphoid organs, which suggests specific resistance of some tissue-resident B-cell subsets. The first model was a transgenic mouse expressing human CD20 and treated with an anti-hCD20 (2H7) monoclonal antibody (mAb). Despite complete depletion of circulating lymph nodes and peritoneal-cavity B-cells, a large fraction of B-cells remained in the spleen, mainly represented by marginal-zone B-cells whose resistance was not related to the dose of hCD20-depleting antibody (5, 6).

Two groups have developed anti-mouse CD20-depleting mAbs (6, 7). Using one of them (MB20-11 antibody, Ig2a), Hamaguchi et al. suggested that despite extensive depletion of all B-cell subsets in blood, spleen, or lymph nodes, the peritoneal-cavity provided a niche for B1 and conventional B lymphocytes, which were resistant to the anti-CD20 treatment. Of note, inflammation resulting in the migration of effector cells, mainly monocytes, facilitated the depletion of these peritoneal B-cells (8). Using another mouse antibody (18B12), one group reported the persistence of germinal-center B-cells in spleen on injection of the depleting antibody at the peak of the immune response (9). In both models (anti-human or mouse CD20 mAbs), lupus-prone mice showed incomplete B-cell depletion in secondary lymphoid tissues, which was explained by a defect in monocyte/neutrophil IgG-mediated phagocytosis (6, 7, 10, 11). These results suggested that specific resistance to B-cell depletion could occur under auto-immune conditions, the failure of such therapies being related to incomplete elimination of auto-reactive B-cell clones in secondary lymphoid organs.

In humans, as in the mouse, CD20 is expressed from pro-B to memory B-cells. Rituximab achieves almost complete peripheral B-cell depletion, with minimal numbers of CD19^+^ cells being detectable in blood within 6 months after treatment. Residual circulating CD19^+^ cells are mainly IgA plasmablasts, as documented by high-sensitivity flow cytometry (12). These cells were suggested to originate from mucosal tissues where they would have been spared from the B-cell depletion, but their presence was not associated with poorer response to treatment in the disease studied, rheumatoid arthritis.

**Figure 1:**
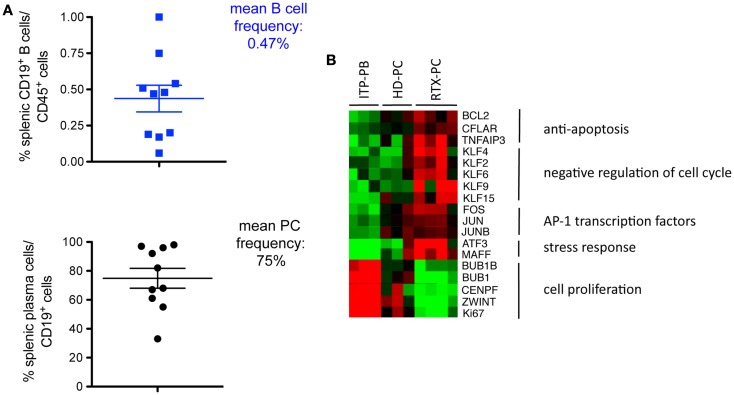
**Long-lived plasma cells in the spleens of ITP patients receiving rituximab**. **(A)** Residual B-cells resisting rituximab treatment in spleens from ITP patients (CD19^+^, % of CD45^+^ spleen cells, upper part) are, for the most part, plasma cells, identified as CD19^+^CD20^−^CD24^−^CD27^+^ CD38^high^ (lower part) (10 patient’s samples analyzed) (13). **(B)** Transcriptomic analysis of splenic plasma cells from four ITP patients receiving rituximab (RTX-PC) reveals a long-lived expression profile as compared with plasmablasts from three ITP patients not treated with rituximab (ITP-PB), and plasma cells from three healthy controls (HD-PC). Heatmap clustering of selected genes classified as anti-apoptosis factors, negative regulators of cell cycle, transcription factors of the AP1 family, stress response genes, and cell proliferation control and marker genes. Genes were selected from the supervised comparison of RTX-PC vs. ITP-PB (609 probes with a fold change >4 or <0.25, and *P* < 0.05). Columns represent individual samples, rows specific gene probes, with upregulated genes in red and downregulated genes in green (twofold scale) (13).

We recently reported marked B-cell depletion in spleens up to 6 months after treatment with rituximab in ITP patients (see below), with only about 0.5% of residual CD45^+^CD19^+^ B-cells, mainly plasma cells (13) (Figure 1A). Thus, in humans, the spleen is not the site of germinal-center or marginal-zone rituximab-resistant B-cells, even in auto-immune conditions.

## B-Cell Depletion in ITP Induces Differentiation into Long-Lived Plasma Cells

Immune thrombocytopenia is an acquired bleeding disorder mediated by pathogenic autoantibodies that enhance platelet destruction and limit their production. The major target of these autoantibodies is the platelet membrane glycoprotein IIb–IIIa (GpIIbIIIa, integrins alpha2b, and beta3), but other glycoproteins can be involved (e.g., GPIb-IX) (14). The spleen is the major site of platelet destruction and is also considered the main site of auto-antibody production, thus seemingly containing all the players required to perpetuate the auto-immune reaction (15). Accordingly, for decades, splenectomy has been the “gold standard” of second-line therapy, resulting in a durable disease cure in two-thirds of patients.

We observed, as did others, that the spleen of ITP patients was the site of an intense B-cell response, with a considerable expansion of short-lived plasmablasts and active germinal-center reactions (13, 16). As described by many authors, plasmablasts generated during a T-dependent response in non-auto-immune conditions will migrate to the bone marrow and differentiate into short-lived plasma cells. Some of these short-lived plasma cells differentiate into long-lived plasma cells (LLPCs) and reside in this niche for a variable length of time. These cells, which represent only 0.5% of mononucleated cells in bone marrow, constitutively secrete antibodies and sometimes persist for decades in humans (17). Differentiation toward LLPCs was described in secondary lymphoid tissues at the site of an immune reaction. Also, from an auto-immune genetic mouse model (NZB/W, lupus-prone), the inflamed environment generated by an auto-immune disease was suggested to be a niche for LLPCs, the cells further perpetuating the local inflammation (17, 18). This observation raised the question as to whether the spleen could similarly represent a site for plasma cell persistence in ITP.

Splenectomy performed in ITP, as standard-of-care treatment or with primary failure of rituximab, provides a unique opportunity to study splenic plasma cells in different settings. We compared the transcriptomic profile of splenic plasma cells in healthy subjects and ITP patients receiving or not rituximab. Most splenic plasma cells in rituximab-receiving patients expressed a program similar to that of bone marrow LLPCs (13). They overexpressed anti-apoptotic factors (*BCL2, CFLAR, TNFAIP3*), negative regulators of the cell cycle, among which are multiple members of the Krüppel-like factor family (*KLF2, KLF6, KLF9, KLF15*). Transcription factors of the AP1 family (*FOS, JUN*, and *JUNB*) were also upregulated, as were genes involved in the unfolded protein response (*ATF3, MAFF*) (Figure 1B). By contrast, plasmablasts, found in ITP patients not receiving rituximab, showed a cell proliferation profile characterized by the expression of positive regulators or markers of the cell cycle (*BIRC5, CENPF, BUB1, BUB1B, ZWINT, CDC6, MKI67*). Surprisingly, analysis of normal plasma cells and plasma cells from ITP patients revealed an intermediate gene expression profile between short-lived plasmablasts and LLPCs. To determine whether this observation was due to a mixture of two populations, we analyzed plasma cells from healthy donors and ITP patients at the single-cell level. Unexpectedly, most cells expressed an intermediate profile between the two populations, with <15% of splenic plasma cells displaying a long-lived signature (13).

These results raised several questions. First, they suggested that an auto-immune inflammatory milieu *per se* does not systematically create a niche for LLPCs (17); second, that LLPCs may only be a minor component of the plasma cell pool in the normal spleen; third, that the B-cell depletion could induce a new microenvironment allowing for short-lived splenic plasma cells to differentiate into long-lived ones. Remarkably, the presence of LLPC in the spleen has mainly been documented after B-cell depletion in mice (through irradiation and anti-CD20 treatment), a situation that, like with rituximab treatment, may have artificially induced their differentiation *in situ* (19, 20). Moreover, some of these post-rituximab splenic LLPCs secreted anti-platelet antibodies, thus explaining the treatment failure.

## Plasma Cell Lifespan: The Essential Role of the Microenvironment

The persistence of LLPCs depends on signals from the microenvironment, including direct cell–cell contact and production of survival factors. Many different factors and cells have been described, both in mice and humans, as being essential for the survival of LLPCs in bone marrow; such factors include the cytokines a proliferation-inducing ligand (APRIL) and interleukin 6 (IL-6) and the chemokine CXCL12 secreted by stromal cells, which attracts CXCR4-positive plasma cells (21). In mice, megakaryocytes and eosinophils are involved in the survival of LLPCs in their bone marrow niche (22). LLPCs express very late antigen 4 (VLA-4) and lymphocyte function-associated antigen 1 (LFA-1), as well as CD44 and P-selectin glycoprotein ligand 1 (PSGL-1), all involved in their survival. However, we still do not know what triggers the differentiation of a small number of short-lived plasma cells into LLPCs as they settle into the bone marrow.

APRIL and B-cell activating factor (BAFF) are two key cytokines that belong to the tumor necrosis factor family: they share receptors such as transmembrane activator and calcium-modulator and cyclophilin ligand interactor (TACI) and B-cell maturation antigen (BCMA); BAFF can also signal through BAFF receptor (BAFF-R), and APRIL can bind to heparan sulfate proteoglycans. BAFF-R is mainly expressed on immature and naive cells, whereas plasmablasts and plasma cells express TACI and BCMA, the latter markedly upregulated on bone marrow LLPCs (23). APRIL is probably the key survival factor for plasma cells, but various gene inactivation experiments have suggested, at least in the mouse, that BAFF and APRIL may substitute for each other in plasma cell maintenance (24). In addition to a survival function, these two molecules may play a role in differentiation from plasmablasts to plasma cells and possibly LLPCs.

With *in vitro* culture of splenic cells, we observed increased BAFF level in the medium from rituximab-treated spleen samples with B-cell depletion as compared to ITP spleens not exposed to rituximab, with no difference in APRIL secretion. Moreover, preliminary experiments showed that normal plasma cells survived better in *in vitro* cultures in the presence of BAFF (13). Indeed, increased BAFF concentration has been reported to likely be a direct consequence of B-cell depletion, its accumulation resulting from a lack of consumption by naive B-cells (25). Interestingly, CD138, a heparan sulfate, has been proposed to bind APRIL and concentrate it in the plasma cell niche (26). CD138 is a specific marker of LLPCs in bone marrow, but human splenic plasma cells are negative for surface expression of CD138 (27), while expressing it at the mRNA level (13). Therefore, BAFF may have a preferred survival role in the context of the splenic plasma cell microenvironment and a specific role in plasma cell differentiation (26, 28).

**Figure 2:**
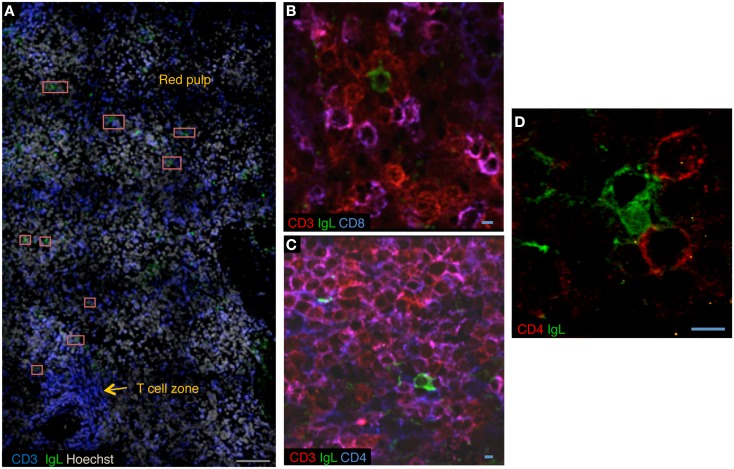
**A CD4^+^ T-cell niche for long-lived plasma cells in rituximab-treated spleens**. **(A)** Confocal microscopy of sections of rituximab-treated spleen stained with anti-CD3 (blue), anti-kappa/lambda light chains (green), and Hoechst (cyan). Plasma cells are located adjacent to the T-cell zone and in red pulp. Red squares mark plasma cells. **(B,C)** Confocal microscopy of sections of rituximab-treated spleen stained with anti-kappa/lambda light chains (green), anti-CD3 (red), and anti-CD8 (blue) **(B)**, or anti-CD3 (red) and anti-CD4 (blue) **(C)**. **(D)** Sections of rituximab-treated spleen stained with anti-kappa/lambda light chains (green) and anti-CD4 (red). Data are representative of three spleen samples. Scale bars: gray 100 μm, blue 5 μm.

The cellular components of the splenic plasma cell niche are not well established. In mice, basophils have been proposed to play a role in plasma cell survival by secreting BAFF and APRIL (29). Stromal cells in the human spleen secrete IL-6 (27). The B-cell depletion induced by rituximab provided us with a unique opportunity to investigate the splenic microenvironment of LLPCs by confocal microscopy. Plasma cells were unambiguously identified as cells strongly expressing kappa/lambda light chains and not CD20. We observed plasma cells in the periphery of the T-cell zone and in the red pulp (unpublished data, Figure 2A). Unexpectedly, in the three spleen samples studied, approximately 20% of plasma cells co-localized with CD3^+^ T cells. In most cases, we observed interaction of one plasma cell with two or three T cells, either CD4^+^ (Figures 2B–D) or possibly double-negative T cells (data not shown). In a co-culture system, CD3^+^CD4^+^ T cells isolated from rituximab-treated spleens did not increase the survival of autologous plasma cells [data not shown and Ref. (30)], which may suggest distinct roles for cells involved in direct contact, providing retention in a defined environment, and cells in close proximity, producing survival signals. A more thorough analysis of the splenic plasma cell niche after rituximab-induced B-cell depletion is in progress.

## Interfering with Auto-Reactive Plasma Cell Persistence, a Future Goal in Auto-Immune Diseases

We demonstrated that B-cell depletion in ITP induced the differentiation of short-lived auto-immune plasma cells into long-lived ones in the spleen. This observation might be of general relevance. In fact, many immunosuppressive and/or biological agents largely used in auto-immune diseases (cyclophosphamide, mycophenolate mofetil, steroids) confer various degrees of B-cell depletion. Of note, rituximab failure in specific diseases such as lupus was often documented in conditions treated with various depleting treatments, which suggests that differentiation into LLPCs was already achieved. In contrast, interfering with the plasma cell survival niche at the time of B-cell depletion might greatly improve the success of these treatments. One first target could be BAFF, because increased level of BAFF accompanies B-cell depletion. Belimumab (monoclonal anti-BAFF antibody) has been approved for the treatment of lupus, with conflicting results (31). Thus, combined anti-CD20 and anti-BAFF therapy might be a first way to interfere with plasma cell persistence. Identification of key cytokines and accessory cells that promote plasma cell differentiation and/or constitute the plasma cell niche in B-cell depleted environments, both in mouse models and in human tissues, may allow for the development of new strategies in antibody-mediated auto-immune diseases.

## Conflict of Interest Statement

The authors declare that the research was conducted in the absence of any commercial or financial relationships that could be construed as a potential conflict of interest.
